# Environmental Factors Drive the Changes of Bacterial Structure and Functional Diversity in Rhizosphere Soil of *Hippophae rhamnoides* subsp. *sinensis* Rousi in Arid Regions of Northwest China

**DOI:** 10.3390/microorganisms13081860

**Published:** 2025-08-08

**Authors:** Pei Gao, Guisheng Ye, Siyu Guo, Yuhua Ma, Yongyi Zhang, Sixuan Sun, Lin Guo, Hongyuan San, Wenjie Liu, Qingcuo Ren, Shixia Wang, Renyuan Peng

**Affiliations:** 1 Agriculture and Animal Husbandry College, Qinghai University, Xining 810016, China; y200954000466@qhu.edu.cn (P.G.); qhxjygs@163.com (G.Y.); siyu_guo1005@outlook.com (S.G.); zhangyy.1213@outlook.com (Y.Z.); sunsixuan6757@163.com (S.S.); 18697158577@163.com (L.G.); 18997410490@163.com (H.S.); lwj2324222641@163.com (W.L.); 17709743937@163.com (Q.R.); wsx1171226@163.com (S.W.); 13672776110@163.com (R.P.); 2 Academy of Animal Husbandry and Veterinary Sciences, Qinghai University, Xining 810016, China

**Keywords:** *Hippophae rhamnoides* subsp. *sinensis* Rousi, geographical pattern, pH, function prediction, rhizosphere soil

## Abstract

*Hippophae rhamnoides* subsp. *sinensis* Rousi has high ecological and medicinal value, and it is an important plant resource unique to the arid regions of Northwest China. Exploring the influence of climate characteristics and soil factors on the composition, diversity, and function of the rhizosphere bacterial community of Chinese seabuckthorn is of great value for developing and popularizing characteristic plant resources in the arid regions of Northwest China. In this study, the rhizosphere soil of 13 Chinese seabuckthorn distribution areas in the northwest of China was taken as the research object, the bacterial community map was constructed based on 16S rRNA gene high-throughput sequencing technology, and the species abundance composition, structural diversity, molecular co-occurrence network, and phylogenetic investigation of communities by reconstruction of unobserved states (PICRUSt), as well as the function of rhizosphere soil bacterial community, were systematically studied. Combined with Mantel test and redundancy analysis (RDA), the key habitat factors driving the rhizosphere soil bacterial community structure of Chinese seabuckthorn were explored. The results showed that: (1) The number of amplicon sequence variants (ASVs) in rhizosphere soil bacterial community of Chinese seabuckthorn was the highest in S2(3072) and the S12(3637), and the lowest in the S11(1358) and S13(1996). The rhizosphere soil bacterial community was primarily composed of Proteobacteria, Actinobacteriota, and Acidobacteriota. Except for the S6 and S11 habitats, the dominant bacterial genera were mainly *Achromobacter*, *Acidobacter* (*RB41*), and *Sphingomonas*. (2) The α and β diversity of rhizosphere soil bacterial communities of Chinese seabuckthorn across 13 distribution areas were significantly different. The number of operational taxonomic units (OTUs), Ace index, and Chao 1 index of soil bacterial community in the S12 distribution area are the highest, and they are the lowest in S11 distribution area, with significant differences. The aggregation of soil bacterial communities in the S5 and S10 distribution areas is the highest, while it is the lowest in the S6 and S11 distribution areas. (3) PICRUSt function classification of soil bacteria showed that Metabolism and Genetic Information Processing functions were the strongest across all distribution areas, with S10 exhibiting higher functional capacity than other areas and S11 showing the weakest. (4) Cluster analysis revealed that soil bacteria across the 13 distribution areas were clustered into two groups, with S10 and S12 distribution areas as one group (Group 1) and the remaining 11 distribution areas as another group (Group 2). (5) Redundancy analysis revealed that pH was the key soil environmental factor driving the rhizosphere soil bacterial community α-diversity of Chinese seabuckthorn, followed by altitude (ALT) and soil water content (SWC). In summary, Chinese seabuckthorn prefers neutral to alkaline soils, and environmental factors play an important role in driving bacterial diversity, community structure, functional profiles, and co-occurrence networks in rhizosphere soil of Chinese seabuckthorn.

## 1. Introduction

*Hippophae rhamnoides* subsp. *sinensis* Rousi, commonly known as Chinese seabuckthorn, acid thorn, black thorn, and acid thorn willow, is a deciduous shrub or small tree of the genus *Hippophae* in the family Elaeagnaceae [1]. The species is naturally distributed in China, Nepal, and Bhutan, with the main distribution areas in China covering Qinghai, Gansu, and Xinjiang provinces and sporadic distributions in Sichuan, Tibet, and Hebei [2,3,4]. Its vertical growth zone is located between 1200 and 4800 m above sea level, and its typical habitats include ecologically fragile areas such as sunny ridges, valley terraces, river valley terraces, and slopes. As a typical pioneer plant, Chinese seabuckthorn demonstrates remarkable ecological roles: Its root nodules effectively fix atmospheric nitrogen, significantly increasing soil nitrogen content. Meanwhile, the plant’s well-developed root system forms a subterranean “network structure”, which, combined with litter decomposition that enhances organic matter accumulation, effectively improves soil structure. This composite ecological function of “nitrogen fixation–soil improvement” establishes Chinese seabuckthorn as a key tree species for ecological restoration in the arid region of Northwest China, holding irreplaceable ecological value in restoration projects [1,4,5,6]. In the field of ethnic medicine, Chinese seabuckthorn is an important plant resource in the Tibetan medicine system. According to the Tibetan medicine classic Yue Wang Yao Zhen, which was written in the 8th century, its fruit has three functions: invigorating the spleen and promoting digestion, relieving cough and eliminating phlegm, and promoting blood circulation and removing blood stasis [7]. Modern pharmacological research further reveals that Chinese seabuckthorn fruit is rich in active ingredients such as flavonoids, vitamins, and unsaturated fatty acids, and the fruit extract has the characteristics of reducing blood lipid, protecting gastric mucosa, relieving inflammation and asthma, and enhancing human immunity [8]. Its cooperative development model of ecological value and economic value provides a typical example for desertification control and regional sustainable development.

In the soil microbial community, rhizosphere bacteria, as the core functional group, form a precise symbiotic network with plant roots, and their community structure dynamics directly participate in the regulation of plant life cycle processes [9]. Such microorganisms enhance the environmental adaptability of host plants by reshaping rhizosphere microecology and driving the biogeochemical cycling of elements [10]. Studies have shown that vegetation restoration can induce changes in the physical and chemical properties of soil, thereby affecting the composition of soil bacterial communities [11]. Vegetation planting in mining areas can reduce soil pH, increase total nitrogen (TN), total phosphorus (TP), and total potassium (TK) contents, and affect the relative abundance of Actinobacteria, Proteobacteria, Gemmatimonadetes, and Bacteroidetes in soil bacterial communities [12,13]. Concurrently, another study found that this interaction exhibits bidirectional regulatory characteristics; that is, the community composition and abundance of microorganisms can also regulate the growth and development of plants reciprocally, enhance the resistance of plants, and affect the distribution of plants [14]. For instance, nitrogen-fixing bacteria in the rhizosphere of *H. rhamnoides*, predominantly belonging to the Actinobacteriota phylum, can convert atmospheric nitrogen into bioavailable forms, providing precursors for amino acid and protein synthesis and constituting a critical pathway for plant nutrient acquisition [15]. Furthermore, certain bacterial symbionts can enhance the activity of antioxidant protective enzymes in plants and induce the production of corresponding secondary metabolites, thus promoting plant resistance to harsh external environments and favouring the expansion of their distribution areas [16].

The extreme environmental characteristics of the Tibetan Plateau, the highest geographic unit in the world, include strong UV radiation, low oxygen partial pressure, dramatic diurnal temperature differences, and significant differences in hydrothermal gradients [17]. This special habitat drives the endemic plants in the plateau and rhizosphere microbial communities to form a symbiotic adaptation system, in which soil bacteria play a core role in maintaining the plant-soil system by participating in key ecological processes such as nutrient transformation and stress tolerance [18]. In recent years, international academic circles have carried out multi-dimensional research on typical plant rhizosphere microbial groups: Wu et al. revealed the dynamic law of rhizosphere bacterial community in poplar plantation with forest age succession and found that available phosphorus is the key soil factor driving community structure variation [19]; Imam team systematically evaluated the regulation effect of biocontrol agent of *Bacillus amyloliquefaciens* on crop rhizosphere microecology through potato rhizosphere microbial analysis [20]; research on Chinese seabuckthorn has analyzed the biogeographic patterns of its rhizosphere mycobiome across different distribution areas from the perspective of rhizosphere fungal geography and identified a core microbiome composed of 10 core fungal phyla [21].

Although Chinese seabuckthorn serves as a key ecological functional species in the arid region of Northwest China, and significant progress has been made in studying its pharmacological active components, population ecology, and rhizosphere fungal community structure, there remains a substantial knowledge gap in understanding its rhizosphere bacterial microecosystem [3,8,21,22]. In order to reveal the key environmental driving factors driving the spatial differentiation of rhizosphere bacterial communities of Chinese seabuckthorn, identify core bacterial taxa with ecological adaptation significance, and explore potential functional microbial resources, wild Chinese seabuckthorn from 13 typical distribution areas in the arid regions of Northwest China was took as the research object, the species composition abundance, diversity distribution pattern, and functional genes of the rhizosphere bacterial community of this species were identified by integrating the climatic characteristics, soil physicochemical factors, and high-throughput sequencing technology. The findings will provide a microbiological perspective for analyzing the environmental adaptation mechanism of Chinese seabuckthorn and provide theoretical support for the ecological restoration and sustainable cultivation of this species. Notably, these insights will facilitate the development of bio-fertilizers and stress-resistant agents based on rhizosphere growth-promoting bacteria.

## 2. Materials and Methods

### 2.1. Sampling Site Description of Chinese Seabuckthorn

**Figure 1:**
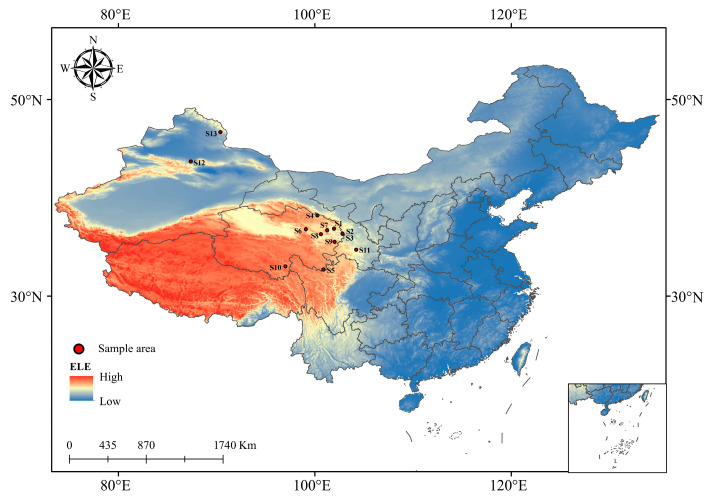
Geographic location of the 13 sampling sites. Note: This figure was created using the standard map (approval number: GS (Beijing, China) No. 1061 (2022) downloaded from the Standard Map Service Website of the China Bureau of Surveying and Mapping Geographic Information. The base map remains unaltered. The darker the blue on the map, the lower the altitude; closer to red means higher altitude.

From mid-July to early August 2023, the research team conducted rhizosphere soil sampling of Chinese seabuckthorn in the Northeastern Qinghai-Tibet Plateau and its adjacent areas, encompassing 13 typical sampling points in Qinghai, Gansu, and Xinjiang provinces (autonomous regions). The specific sampling regions encompass Huzhu Tuzu Autonomous County (S1), Minhe Huizu and Tuzu Autonomous County (S2), Ledu District (S3), Qilian County (S4), Banma County (S5), Wulan County (S6), Huangyuan County (S7), Gonghe County (S8), Tongren City (S9), and Yushu City (S10) in Qinghai Province; Zhang County (S11) in Gansu Province; and Urumqi County (S12) and Qinghe County (S13) in the Xinjiang Uyghur Autonomous Region. All sampling regions exhibit typical plateau continental climate characteristics: annual mean precipitation ranges from 20 mm to 160 mm, annual mean temperature varies between −0.5 °C and 6.5 °C, annual mean evaporation reaches 1200–2500 mm, annual sunshine duration persists at 2100–2800 h, and diurnal temperature variation can attain 20–25 °C [21]. The associated plant communities of *Hippophae rhamnoides* subsp. *sinensis* populations in the surveyed regions exhibit common compositional characteristics, including grass species such as *Elymus nutans* and *Poa pratensis*, alongside typical alpine herbs like *Astragalus polycladus*, *Saussurea arenaria*, *Gentiana crassicaulis*, and *Lancea tibetica*. These species collectively form a specialized plant community structure adapted to extreme habitats. The geographical locations of the 13 sampling sites are illustrated in Figure 1, with detailed geographic coordinates and climatic parameters provided in Table 1.

### 2.2. Rhizosphere Soil Sampling of Chinese Seabuckthorn

Collecting rhizosphere soil during flowering and fruiting period of Chinese seabuckthorn in China. In 13 target sampling areas (Table 1), 9 repeated sampling points are set in each area, the sampling points are distributed in an S-shaped route, and the interval between adjacent points is 8 m. The Riley and Barber method [23] was strictly followed for rhizosphere soil separation at each sampling site: the Chinese seabuckthorn root system was first dug intact, and loose soil was naturally dislodged by gentle shaking (Non-rhizosphere soil), followed by meticulous collection of soil particles still attached to the root surface (rhizosphere soil) using sterile brushes. In each region, the rhizosphere soil of three adjacent sampling points was mixed equally, and, finally, 39 ecologically representative soil samples (13 areas × 3 samples/area) were obtained, and the sampling depth was uniformly controlled at 0–50 cm in the topsoil layer.

The sample treatment followed the dual-track preservation strategy: fresh rhizosphere soil is immediately divided into two sub-samples after being passed through a 2 mm sieve. One sub-sample was placed in a sterile ziplock bag and stored at −20 °C for soil physicochemical property analyses; the other sub-sample was dispensed into a 50 mL centrifuge tube, snap-frozen in liquid nitrogen, and then transferred to an ultra-low-temperature refrigerator (MDF-86V588D, Zhongke duling, Hefei, China) at −80 °C for preservation and dedicated to subsequent microbiomics studies [24]. Soil physicochemical properties were measured strictly following Bao Shidan’s “Soil Agrochemical Analysis” (3rd edition) [25]. The detection methods for each indicator are as follows: soil water content (SWC) was determined by the drying method (LY/T1213-1999); pH value was measured using the potentiometric method (soil–water ratio 1:2.5); electrical conductivity (SEC) was assessed via the 15% H_2_O_2_ dry weight method (LY/T1251-1999); soil organic matter (SOM) was quantified using the potassium dichromate volumetric method; total nitrogen (STN) was measured by the Kjeldahl method; total phosphorus (STP) content was determined through the alkali fusion–molybdenum–antimony spectrophotometric method; total potassium (STK) content was analyzed using the alkali fusion–flame photometric method; available nitrogen (SAN) was assessed via the alkaline diffusion method; available phosphorus (SAP) was measured using the molybdenum–antimony colorimetric method; and available potassium (SAK) was determined with a flame photometer. Specific soil physicochemical property indices for the 13 sampling sites are detailed in Table 2.

### 2.3. Soil Bacterial DNA Extraction, PCR Amplification, and High-Throughput Sequencing for Chinese Seabuckthorn

Rhizosphere soil sample (0.5 g) was weighed, and genomic DNA extraction was performed using the DNA extraction kit (NucleoSpin, Merck group, Dueren, Germany), with the extraction process strictly following the kit instructions [26]. DNA integrity was verified via 1% agarose gel electrophoresis, and concentration and purity assessments of DNA were conducted using a nucleic acid analyzer to ensure library construction quality [26]. The quality standards of the DNA samples submitted for inspection were: DNA concentration ≥ 50 ng/μL and volume ≥ 60 μL, DNA purity A260/A280 ratio of 1.8–2.0 (no obvious protein or RNA pollution), and agarose gel electrophoresis shows that the main band is clear and there are no signs of degradation or restriction. The DNA samples that meet the quality inspection standards were sent to a professional sequencing agency (Beijing Novojin Bioinformation Technology Co., Ltd., Beijing, China) to carry out amplicon sequencing for the V4 variable region of bacterial 16S rRNA gene [27]. PCR amplification conditions: A classical primer pair (515F: 5′-GTGCCAGCMGCCGCGGTAA-3′ and 806R: 5′-GGACTACHVGGGTWTCTAAT-3′) was employed [27]. The reaction mixture consisted of 2 μL of 10× buffer, 2 μL of dNTPs (2.5 mmol/L), 0.8 μL each of forward and reverse primers, 0.2 μL of polymerase, 1 μL of template DNA, and ddH_2_O to achieve a final volume of 20 μL. Thermal cycling parameters were optimized as follows: initial denaturation at 95 °C for 3 min; followed by 35 cycles of denaturation at 95 °C for 30 s, annealing at 55 °C for 30 s, and extension at 72 °C for 45 s; and a final extension at 72 °C for 5 min. Amplicons were stored at 4 °C. Post-amplification processing: Qualified products verified by agarose gel electrophoresis were subjected to equimolar pooling and subsequent purification [21,26]. Library construction strictly followed standard protocols: After electrophoresis again, the target band was recovered by using Qiagen gel recovery kit. Libraries were prepared with the NEBNext^®^ UltimaTM II DNA Library Prep Kit (Ipswich, MA, USA), followed by quantification via Qubit and quantitative Q-PCR. Qualified libraries were sequenced on the NovaSeq 6000 platform. Post-sequencing data underwent quality control and chimera removal. Sequences were denoised and filtered (abundance < 5) using the DADA2 plugin in QIIME 2, yielding amplicon sequence variants (ASVs) and feature tables. Then, compare ASVs with the database of Silva138.1 with the classify-sklearn module of QIIEM2 to obtain species information. ASV of Chloroplast and Mitochondria sequences should be removed, and at the same time, the data should be diluted during the analysis to ensure the uniformity of sequencing depth among samples. Sequencing and annotation were completed by Novogene Bioinformatics Technology Co., Ltd. (Beijing, China) [26,27].

### 2.4. Data Analysis

Raw data organization was performed using Microsoft Excel 2019 (Microsoft, Washington, DC, USA), and statistical analysis was conducted with SPSS 25.0 (IBM, New York, NY, USA). Additionally, soil bacterial community visualization analyses—including abundance analysis, diversity analysis, clustering analysis, LEfSe statistical analysis, and PICRUSt functional prediction—were carried out via the Novogene Cloud Platform (https://magic.novogene.com) [21,26,27]. QIIME 2 software (202202) was used to generate rarefaction curves, species relative abundance plots, PCoA plots (Bray-Curtis), and UPGMA clustering trees, as well as to calculate α-diversity indices (OTUs, Shannon index, Simpson index, Pielou index, Invsimpson index, Chao1 index, ACE index, Goods coverage). To further explore differences in community structure between grouped samples, LEfSe statistical analysis was applied to test the significance of differences in species composition and community structure [21,26,27]. PICRUST is a biological information software package for predicting the function of soil microorganisms based on Marker gene (such as 16S rRNA). Through the correlation between amplicon’s annotation results and the corresponding functional database (KEGG), PICRUST software (V2.5.1) was selected to predict and analyze the functions of microbial communities in ecological samples [28]. Correlations between species were calculated using Networkx (version 1.11) to construct molecular network diagrams. R 3.5.2 software was employed to generate correlation heatmaps between environmental factors, soil physicochemical factors, and microbial diversity. Redundancy Analysis (RDA) of environmental factors, soil physicochemical factors, and soil microbial diversity was performed using Canoco 5.0 (Microcomputer Power, New York, NY, USA). 

## 3. Results and Analysis

### 3.1. Changes in the Quality of 16s Sequencing Results and the Number of ASV in Bacterial Communities

**Figure 2:**
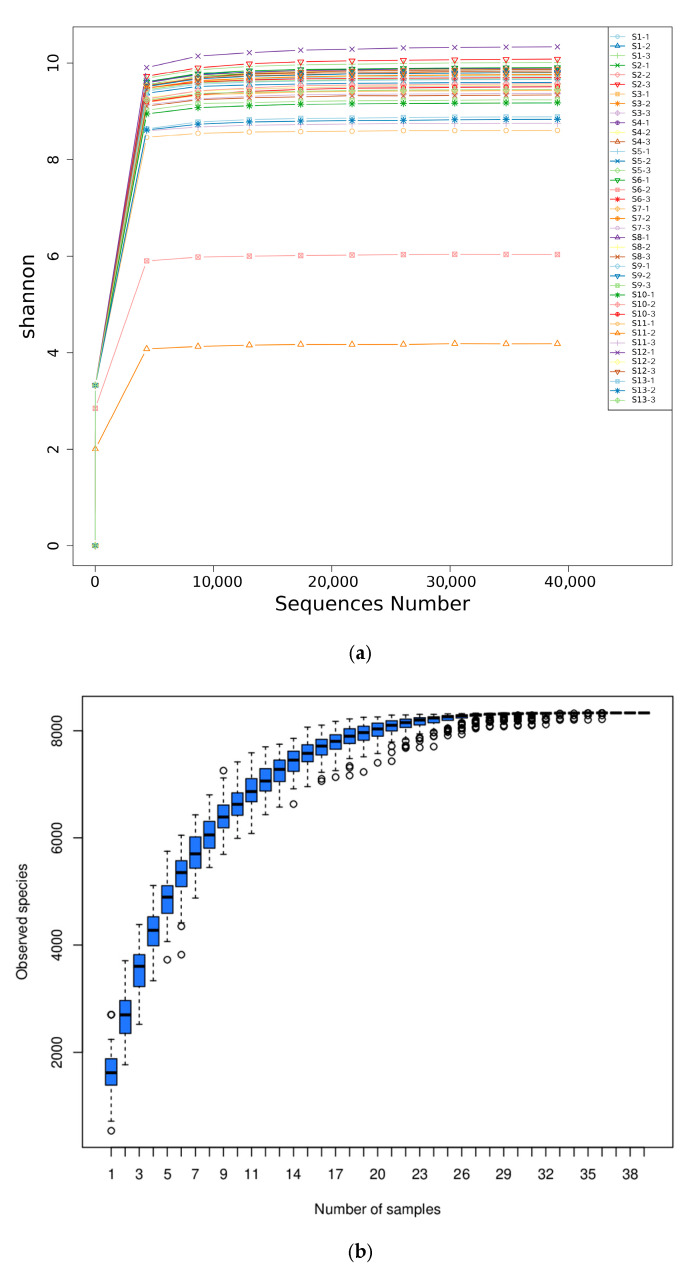

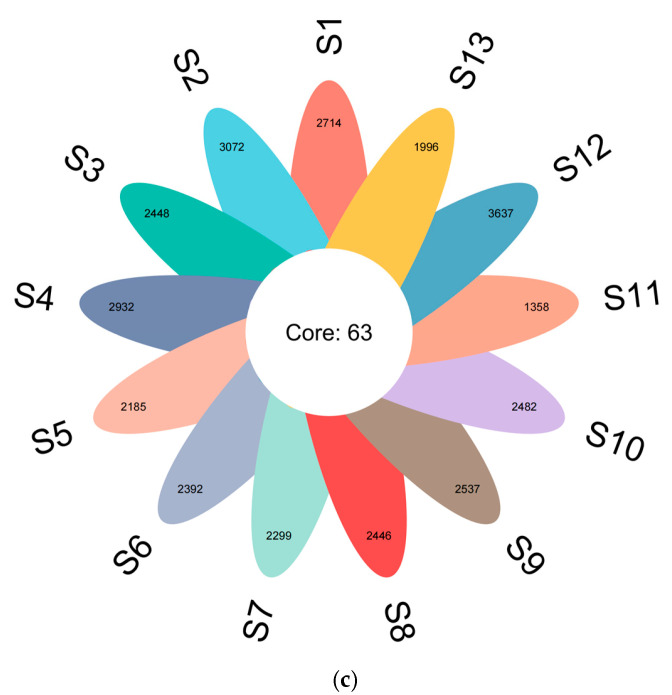
Analysis of sequencing results quality and ASV quantity of 13 distribution regions. (**a**) Dilution curves; (**b**) species cumulative box plots; (**c**) Venn diagrams.

As shown in Figure 2, high-throughput sequencing analysis was carried out on 39 rhizosphere soil samples from 13 distribution regions of Chinese seabuckthorn in the northwest arid area of China. The results showed that with the increasing number of sequencing sequences, the Shannon diversity dilution curves corresponding to 13 geographical distribution areas showed a gradual and gentle trend (Figure 2a). Further analysis (Figure 2b) revealed that when the number of samples collected reached 20, the observed species accumulation curve began to plateau. It represents that the current sequencing data approached the theoretical saturation threshold, and the sequencing depth designed in the experiment is scientific and reasonable, meaning that under these conditions, even if the sequencing scale continues to expand, only a few low-abundance microbial groups can be detected.

Venn diagram analysis was carried out on the bacterial amplicon sequence variants (ASV) of rhizosphere soil samples of Chinese seabuckthorn in 13 distribution regions, and the results were shown in Figure 2c. Among them, the number of ASVs in 13 distribution regions (S1–S13) was 2714, 3072, 2448, 2932, 2185, 2392, 2299, 2446, 2537, 2482, 1358, 3637, and 1996, respectively. The number of ASVs in S12 exhibited the highest, followed by S2, and the number of ASVs in S11 and S13 showed the lowest. In terms of shared ASVs, the number of ASVs shared by the 13 types of distribution regions reached 63, and these shared ASVs represented the core components of the rhizosphere soil bacterial community of Chinese seabuckthorn among different distribution regions.

### 3.2. Changes in Species Composition and Relative Abundance of Soil Bacterial Communities

**Figure 3:**
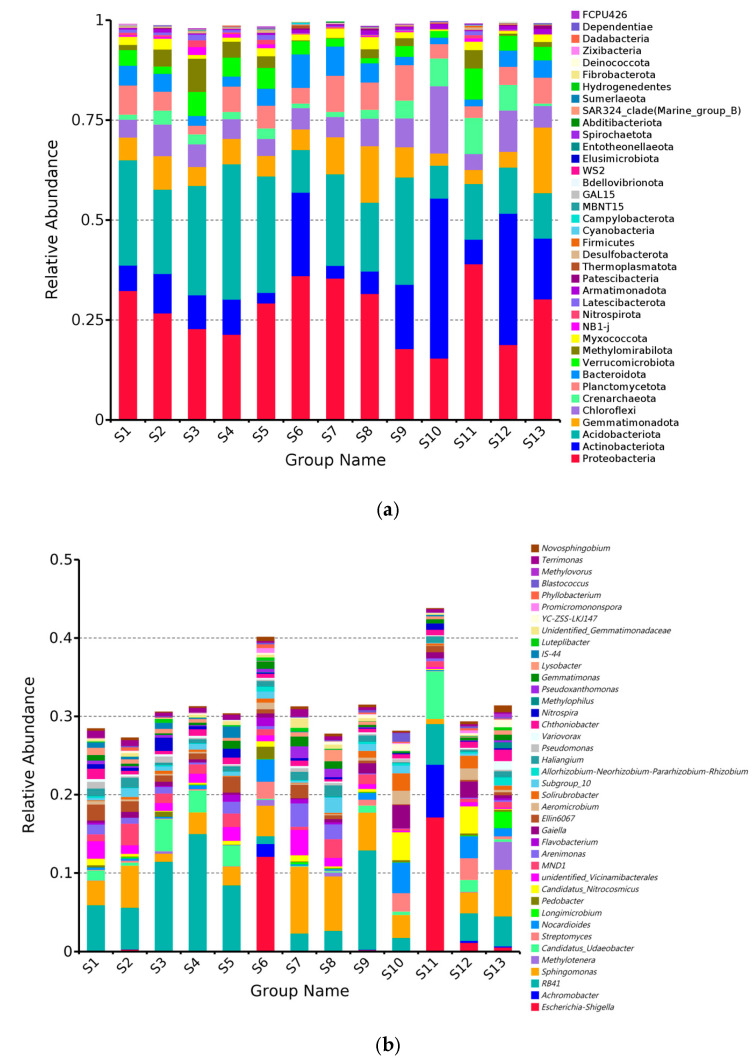
Relative abundance of 13 distribution regions at phylum level and genus level. (**a**) Phylum level; (**b**) genus level.

The relative abundance distribution of the top 40 dominant soil bacterial taxa under the phylum-level classification in 13 Chinese seabuckthorn distribution regions was shown in Figure 3a. Among them, Proteobacteria (15.49–39.08%), Actinobacteriota (2.65–39.95%), Acidobacteriota (8.28–33.83%), Gemmatimonadota (3.02–16.39%), and Chloroflexi (3.98–16.86%) were relatively more abundant and were the core dominant taxa. Soil bacterial phyla in the 13 regions showed spatial heterogeneity, with Proteobacteria dominated in the S1, S2, S5, S6, S7, S8, S11, and S13; Actinobacteriota forming a dominant community in the S10 and S12; and Acidobacteriota specifically enriched in the S3 and S4. The relative abundance of Proteobacteria, Acidobacteriota, and Gemmatimonadota was significantly higher in the remaining 12 regions compared to the S10, while the abundance of Actinobacteriota and Chloroflexi exhibited decreasing abundance trends.

Except for habitats S6 and S11, genera such as *Achromobacter* (0.00–6.73%), *RB41* (0.99–14.92%), *Sphingomonas* (0.65–8.49%), and *Methylotenera* (0.00–3.59%) exhibited relatively high abundances, serving as core dominant genera in the soil bacterial communities. Soil bacterial genera in the 13 distribution categories showed spatial heterogeneity, with *Acetobacter* (*RB41*) dominated in the S1, S3, S4, S5, S9, and S12; *Sphingomonas* significantly enriched in the S2, S7, S8, and S13; and *Nocardioides* constituted the S10 distribution’s characteristically dominant genus. Compared with S11, the relative abundance of *sphingomonas* in the other 12 distribution regions increased significantly, while the abundance of *Achromobacter* exhibited decreasing abundance trends.

### 3.3. LEfSe Analysis of Soil Bacterial Community

**Figure 4:**
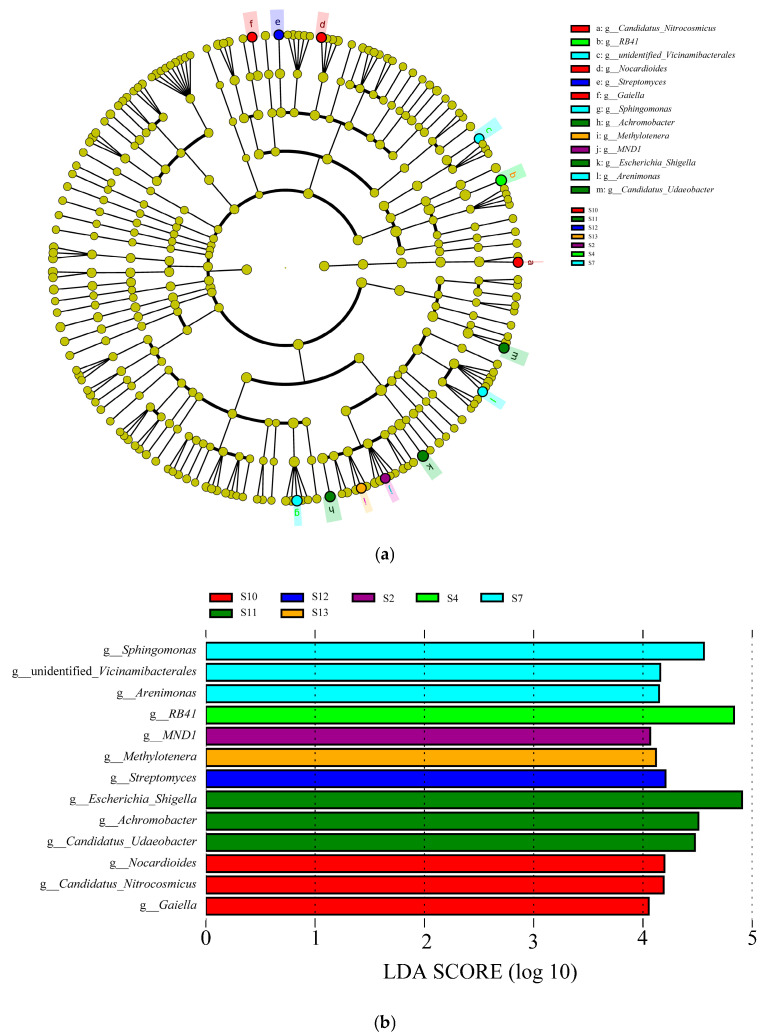
LEfSe analysis of 13 species in the genus level. (**a**) LEfSe analysis (LDA > 4.0); (**b**) LDA value of indicator species (LDA > 4.0).

Through LEfSe analysis (Figure 4), this study successfully identified the key indicator species (LDA threshold > 4.0) driving the difference of bacterial communities in 13 distribution regions, which effectively revealed the core bacterial groups that shaped the variation of community structure. Combining the information in Figure 4a,b, the bacterial communities in seven of all 13 regions (S2, S4, S7, S10, S11, S12, and S13) showed prominent indicator species, with a total of 13 statistically significant biomarkers identified. Specifically, one biomarker was detected for each of S2, S4, S12, and S13, while three biomarkers were detected for each of S7, S10, and S11. At the taxonomic level, the core biomarkers showed a clear differentiation across the distribution regions. The biomarkers with the highest scores in S2, S4, S7, S10, S11, S12, and S13 are *MND1* (4.07), *RB41* (4.83), *Sphingomonas* (4.56), *Nocardioides* (4.20), *Escherichia-Shigella* (4.91), *Streptomyces* (4.21), and *Methylotenera* (4.12), respectively.

### 3.4. Analysis of Soil Bacterial Community Diversity

#### 3.4.1. Analysis of α-Diversity of Soil Rhizosphere Bacterial Community

**Figure 5:**
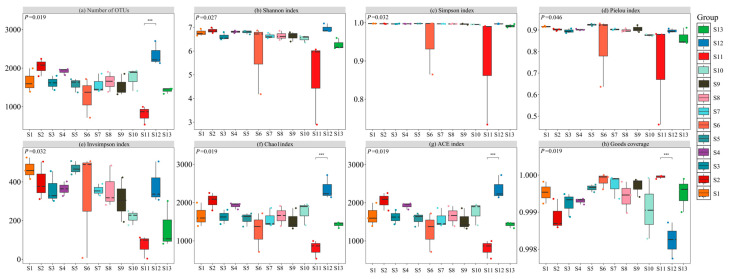
α-diversity of bacterial communities in 13 distribution regions. Note: *** represents *p* < 0.001. (**a**) Number of OUTs, (**b**) Shannon index, (**c**) Simpson index, (**d**) Pielou index, (**e**) Invsimpson index, (**f**) Chao1 index, (**g**) ACE index, (**h**) Goods coverage.

The α-diversity index analysis of rhizosphere soil bacterial communities in 13 regions showed (Figure 5) that there were significant differences across distribution regions *(p* < 0.05). Among them, the number of soil bacterial OTUs and Shannon’s index reached the highest value of 2350.00 and 6.95 in the S12 distribution area, which were significantly higher than those in the S11 (*p* < 0.05), with an increase of 193.75% and 39.56%, respectively. In terms of Simpson index, the regional ranking is S5 > S1 > S2 > S12 > S4 > S7 > S3 > S8 > S9 > S10 > S13 > S6 > S11, and there is no significant difference among other 12 distribution regions, except S11 (*p* > 0.05). The Pielou index of the S5 was as high as 0.92, which was 24.32% higher than that of the S11, and the difference was significant (*p* < 0.05). The Insimpson’s index of S5 was 470.77, which was 5.53 times higher than that of S11, and the difference was significant (*p* < 0.05). In addition, both Ace and Chao1 indices were highest in the S12, at 2366.80 and 2365.04, respectively, which were 195.74% and 195.48% higher than that of S11 (*p* < 0.05). The sequencing coverage of the samples from the 13 distribution regions was in the range of 99.82% to 99.99%, indicating that the sequencing depth had adequately reflected the actual composition of the soil bacterial community. In summary, the S12 significantly enhanced the OTU abundance, diversity indices (Shannon, Ace, Chao1), and evenness (Pielou) of the rhizosphere soil bacteria compared to the S11.

#### 3.4.2. Analysis of Soil Bacterial Community Beta Diversity (PCoA)

**Figure 6:**
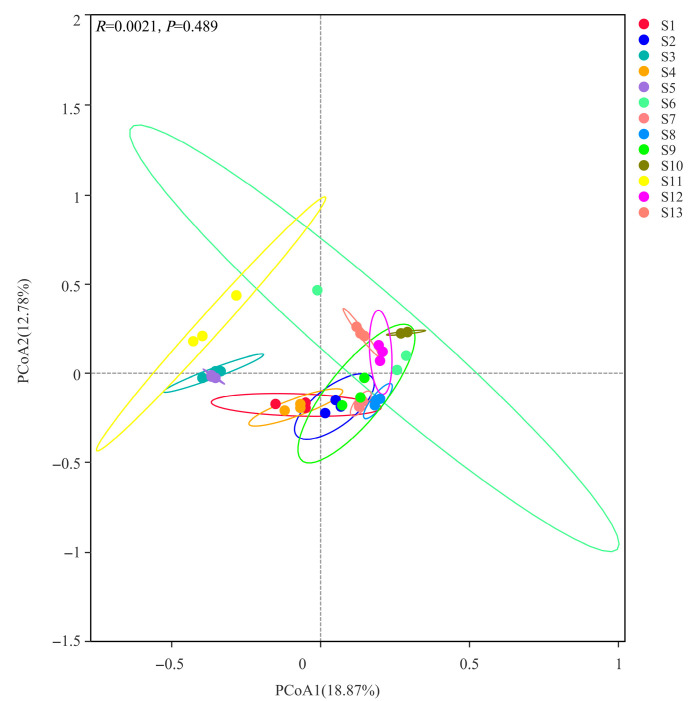
Differences in diversity of bacterial community β (PCoA) in 13 distribution regions.

The results of PCoA analysis based on Bray-Curtis distance (Figure 6) reveal the spatial heterogeneity of rhizosphere soil bacterial community structure in 13 Chinese seabuckthorn distribution regions. The PC1 and PC2 axes explained 18.87% and 12.78% of the community variation, respectively, with a cumulative explanation rate of 31.65%, suggesting that there is a multidimensional regulatory feature in the effect of habitat factors on bacterial community structure. There were no significant differences (*p* = 0.489) in the rhizosphere bacterial communities of Chinese seabuckthorn among the 13 habitats. Additionally, the bacterial communities from different habitats did not form distinct clusters. In terms of community structure aggregation, S6 and S11 showed significant intra-group discrete characteristics, with a high degree of variation in the composition of their bacterial communities. However, the remaining 11 distribution regions showed strong intra-group homogeneity, with the most significant community structure aggregation and the least intra-group variation in S5, S7, S8, S10, and S13.

### 3.5. Co-Occurrence of Soil Bacteria Molecular Network Changes

**Figure 7:**
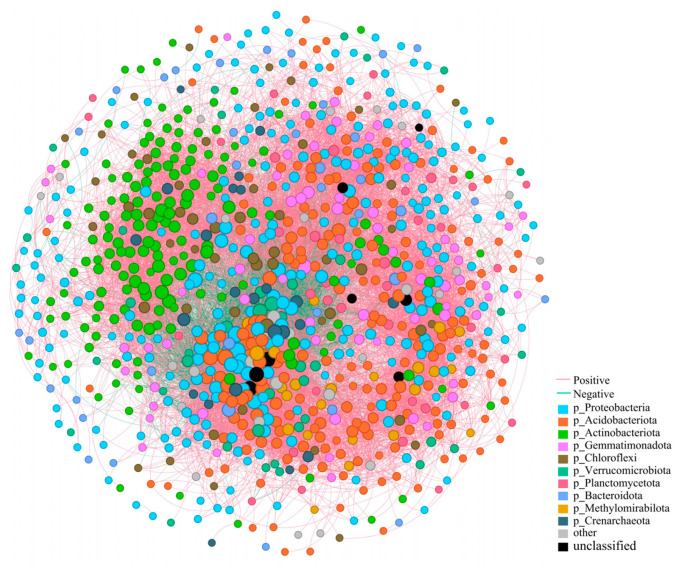
Co-occurrence network diagram of soil bacteria in 13 distribution regions. Note: Node colours and sizes indicate species type and importance; line colours indicate positive or negative correlation, with pink being positive and green being negative.

To elucidate the species co-occurrence patterns of bacterial communities in the rhizosphere soil of Chinese seabuckthorn at the phylum level, a co-occurrence molecular ecological network was constructed based on phylum-level classification (Figure 7). The network comprises 950 bacterial phylum-level nodes and 17,196 ecological association edges. The co-occurrence network exhibits typical complex network topological properties: with an average node connectivity of 36.20, an average path length of only 1.86, and a network diameter of 4.55, indicating intensive interactions among bacterial communities. The clustering coefficient of 0.44 and network density of 0.04 reflect that bacterial phyla maintain both modular aggregation characteristics and global coordination features. The network nodes are predominantly composed of five dominant bacterial phyla: Proteobacteria (27.81%) and Acidobacteriota (21.87%) form the core hubs, while Actinobacteriota (17.20%), Gemmatimonadota (7.22%), and Chloroflexi (5.52%) serve as key components. Notably, 88.19% of the ecological associations in the network showed a positive correlation, and 11.81% showed a positive correlation, indicating that synergistic symbiosis dominates the rhizosphere bacterial community interactions.

### 3.6. Prediction of Soil Bacterial Community Function

**Figure 8:**
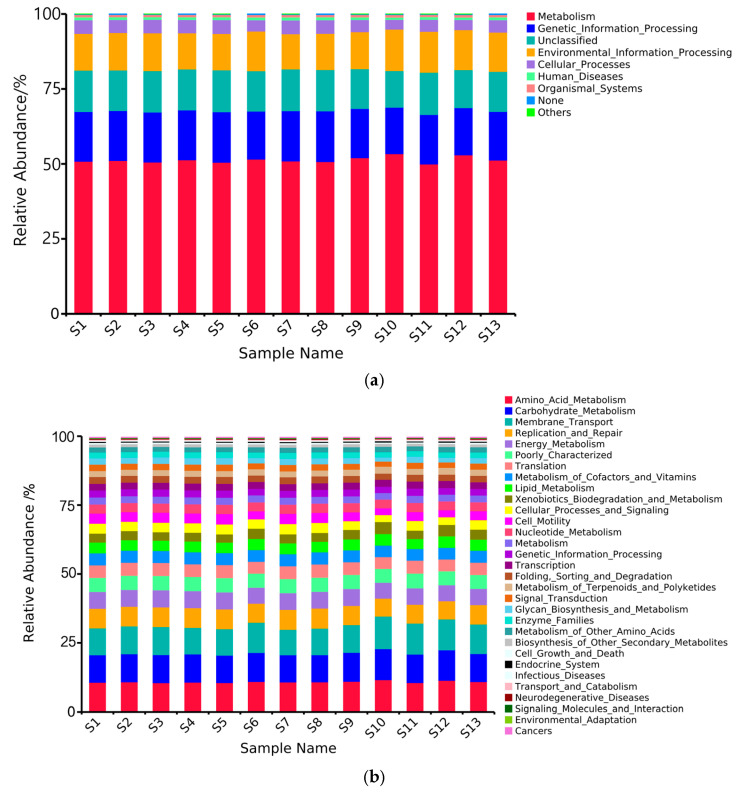
Relative abundance map of PICRUSt function prediction of soil bacteria in 13 distribution regions. Note: (**a**) Level 1; (**b**) Level 2.

Based on the predictive analysis of PICRUSt functional genes, this study revealed the functional composition characteristics (Level 1) of rhizosphere soil bacterial communities in 13 distribution regions of Chinese seabuckthorn (Figure 8a). Functional annotation identified eight major functional modules, with Metabolism, Genetic Information Processing, Unclassified functions, Environmental Information Processing, and Cellular Processes forming the core functional categories. Metabolic function is dominant in all distribution regions, with relative abundance ranging from 49.98% to 53.42%, in which S10 shows significant function enrichment (53.42%), while S11 shows relative lack of function (49.98%). The secondary functional modules demonstrated a tripartite structure: Genetic Information Processing function (15.48–16.90%), Unclassified functions (12.23–14.09%), and Environmental Information Processing (11.73–13.81%) constituted the foundation of community functional diversity. It is noteworthy that compared with S11, the other 12 regions can reduce the abundance of Unclassified bacteria and increase the abundance of Metabolism bacteria.

Based on the predictive analysis of PICRUSt functional genes, this study revealed the functional composition characteristics (Level 2) of rhizosphere soil bacterial communities in 13 distribution regions of Chinese seabuckthorn (Figure 8b). Functional annotation results identified a total of 37 major categories of functional modules, of which Amino_Acid_Metabolism, Carbohydrate_Metabolism, Membrane_Transport, Replication_and_Repair, Energy_Metabolism, Poorly_Characterized, Translation, Metabolism_of_Cofactors_and_Vitamins, Lipid_Metabolism, and Xenobiotics_Biodegradation_and_Metabolism form the core functional categories. The metabolic functions Amino Acid Metabolism, Carbohydrate Metabolism, and Membrane Transport dominated across all 13 distribution regions, with relative abundances ranging from 10.54% to 11.64%, 9.84% to 11.24%, and 9.21% to 11.81%, respectively. Notably, the S10 distribution region exhibited significant functional enrichment, while the S3, S6, and S7 regions showed relatively depleted functional profiles. The secondary functional modules were identified as follows: Replication_and_Repair (6.50~7.28%), Energy_Metabolism (5.77~6.06%), Poorly_Characterized (5.03~5.17%), and Translation (4.27~4.78%). These functions collectively form the foundation of community functional diversity. Notably, compared to the S10 distribution region, the other 12 regions exhibited reduced bacterial abundances in Amino_Acid_Metabolism, Carbohydrate_Metabolism, and Membrane_Transport while showing increased abundances in Replication_and_Repair, Energy_Metabolism, Poorly_Characterized, and Translation functional modules.

### 3.7. Cluster Analysis of Rhizosphere Soil Bacterial Community of Chinese Seabuckthorn in Different Distribution Regions

**Figure 9:**
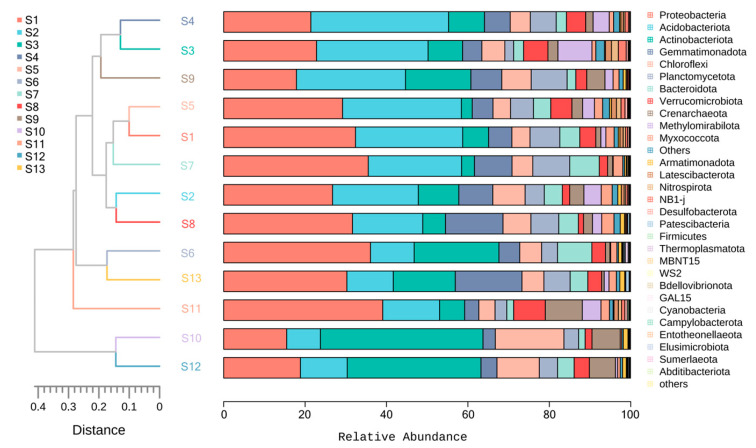
UPGMA cluster tree based on weighted Bray-Curtis distance. Note: The UPGMA cluster tree is on the left, and the relative abundance of soil bacteria at the door level is on the right.

Cluster analysis was performed on rhizosphere soil samples of Chinese seabuckthorn from 13 distribution regions to explore similarities, with results presented in Figure 9. Based on OTU count statistics, UPGMA clustering analysis was conducted using a Bray-Curtis distance matrix, integrating clustering results with phylum-level taxonomic abundance profiles. Clustering of the top 30 bacterial groups by relative abundance at the phylum level partitioned the 13 distribution regions into two major groups: at a clustering distance threshold of 0.40, S10 and S12 initially formed Group 1, while the remaining 11 regions constituted Group 2. Further reducing the clustering threshold to 0.28 induced subgroup differentiation within Group 2: S11 emerged as an independent subgroup, while S1–S9 coalesced into another subgroup. Notably, S1–S9 distribution regions formed a single evolutionary branch on the phylogenetic tree, indicating high similarity in their bacterial community structures. In contrast, the S10, S12, and S11 distributions are located in different evolutionary branches, indicating significant differences in their bacterial community composition.

### 3.8. The Coupling Relationship Between Climate Characteristics, Soil Physicochemical Characteristics Factors, and the Diversity of Rhizosphere Soil Bacterial Community of Chinese Seabuckthorn

#### 3.8.1. Mantel Test Analysis of Climate Characteristics, Soil Physicochemical Characteristics Factors, and Soil Bacterial Community

**Figure 10:**
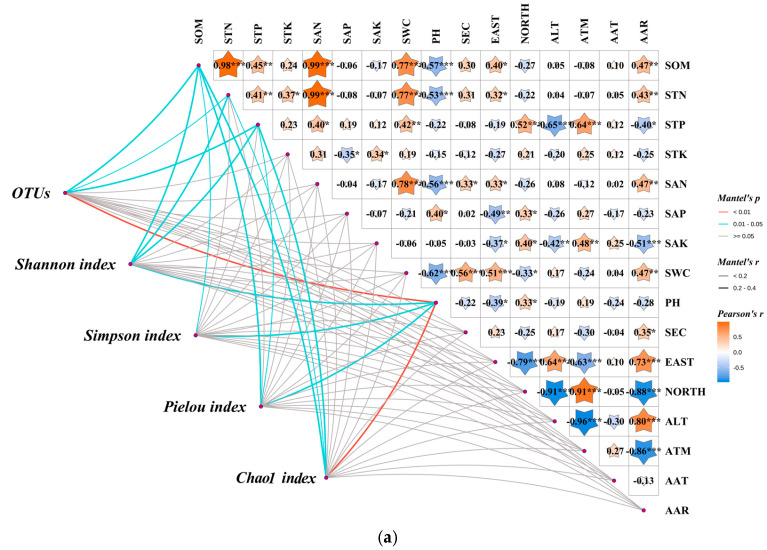

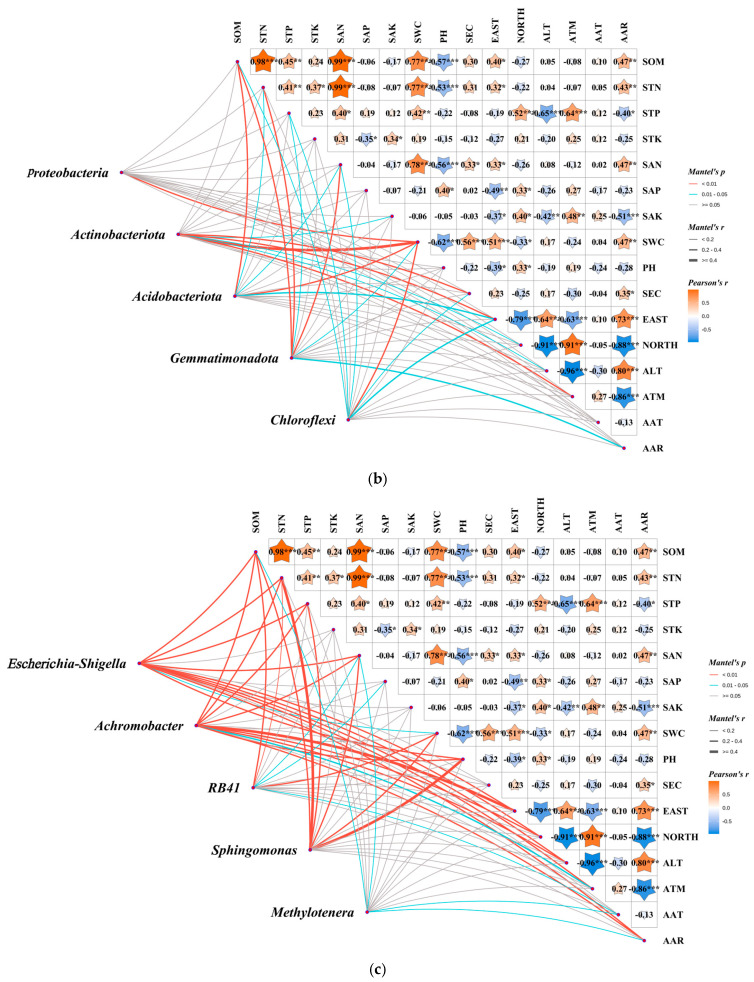
Mantel analysis of climate characteristics, soil physicochemical characteristics, and soil bacterial community. Note: * denotes *p* < 0.05, ** denotes *p* < 0.01, *** denotes *p* < 0.001. (**a**) αdiversity, (**b**) Bacterial phylum level, (**c**) Bacterial genus level.

As shown in Figure 10, Mantel test results revealed the following extremely significant correlations: SOM exhibited an extremely strong positive correlation with STN (*p* < 0.001), and both demonstrated extremely significant positive correlations with SAN and SWC (*p* < 0.001) while showing extremely significant negative correlations with pH (*p* < 0.001). STP displayed extremely significant positive correlations with ATM and NORTH (*p* < 0.001), coupled with an extremely significant negative correlation with ALT (*p* < 0.001). SAN showed an extremely significant positive correlation with SWC and an extremely significant negative correlation with pH (*p* < 0.001). SAK demonstrated an extremely significant negative correlation with AAR (*p* < 0.001). SWC exhibited extremely significant positive correlations with EAST and SEC, along with an extremely significant negative correlation with pH (*p* < 0.001). EAST displayed extremely significant positive correlations with AAR and ALT (*p* < 0.001) while showing extremely significant negative correlations with ATM and NORTH (*p* < 0.001). NORTH exhibited an extremely significant positive correlation with ATM (*p* < 0.001), accompanied by extremely significant negative correlations with AAR and ALT (*p* < 0.001). ALT showed an extremely significant positive correlation with AAR and an extremely significant negative correlation with ATM (*p* < 0.001). ATM demonstrated an extremely significant negative correlation with AAR (*p* < 0.001).

The correlation between bacterial community alpha diversity and soil physicochemical properties/climatic characteristics is presented in Figure 10a. The bacterial community OTUs counts exhibited a highly significant correlation with pH (*p* < 0.01), alongside significant correlations with SOM, STN, and STP (*p* < 0.05). The Shannon index demonstrated significant correlations with SOM, STN, STP, and pH (*p* < 0.05). The Simpson index showed significant correlations exclusively with SOM and pH (*p* < 0.05). The Pielou index displayed significant correlations with SOM, STP, and pH (*p* < 0.05). The Chao1 index presented a highly significant correlation with pH (*p* < 0.01) and significant correlations with SOM, STN, and STP (*p* < 0.05). In the bacterial community, the correlation between the horizontal abundance of the top five phyla and soil physical and chemical characteristics and climate characteristics is shown in Figure 10b. Proteobacteria were highly significantly correlated with SWC (*p* < 0.01). Actinobacteriota exhibited highly significant correlations with SWC, SEC, EAST, and ATM (*p* < 0.01), and significant correlations with SAK, NORTH, and ALT (*p* < 0.05). Acidobacteriota exhibited highly significant correlations with SOM and SWC (*p* < 0.01), and significant correlations with STN, SAN, and EAST (*p* < 0.05). Gemmatimonadota exhibited highly significant correlations with SOM, STN, and SAN (*p* < 0.01), and significant correlations with STP, SWC, and AAR (*p* < 0.05). Chloroflexi exhibited a highly significant correlation exclusively with SWC (*p* < 0.01), and significant correlations with SOM, STP, SAN, and EAST (*p* < 0.05). In the bacterial community, the correlation between the horizontal abundance of the top five genera and soil physical and chemical characteristics and climate characteristics is shown in Figure 10c. *Escherichia-Shigella* exhibited significant (*p* < 0.05) or highly significant (*p* < 0.01) correlations with indicators other than STK, SAP, SEC, and AAT. *Achromobacter* exhibited significant (*p* < 0.05) or highly significant (*p* < 0.01) correlations with indicators other than STK, SAP, SEC, and AAT. *RB41* exhibited highly significant correlations with SOM, STN, and SWC (*p* < 0.01), and significant correlations with SAN, SAP, and AAT (*p* < 0.05). *Sphingomonas* exhibited a highly significant correlation with SOM, STN, STP, SAN, SWC, and pH (*p* < 0.01). *Methylotenera* bacteria exhibited significant correlations with all indicators except SOM, SAN, AAT, and AAR (*p* < 0.05).

#### 3.8.2. RDA Analysis of Climate Characteristics, Soil Physicochemical Characteristics Factors, and Soil Bacterial Community

**Figure 11:**
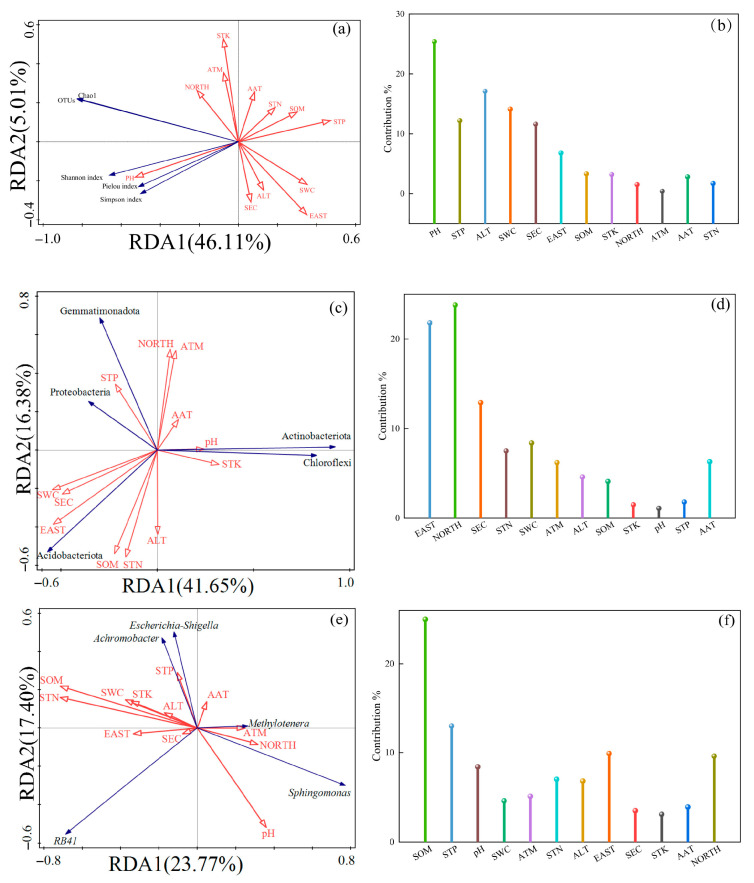
RDA analysis of climate characteristics, soil physicochemical characteristics, and soil bacterial community structure. (**a**) Redundancy Analysis (RDA) between habitat factors and bacterial community alpha diversity, (**b**) Contribution rate of habitat factors to bacterial community alpha diversity, (**c**) Redundancy Analysis (RDA) between habitat factors and the top 5 abundant bacterial phyla, (**d**) Contribution rate of habitat factors to the top 5 abundant bacterial phyla, (**e**) Redundancy Analysis (RDA) between habitat factors and the top 5 abundant bacterial genera, (**f**) Contribution rate of habitat factors to the top 5 abundant bacterial genera.

Based on the results of Redundancy Analysis (RDA) (Figure 11), the relationship between the diversity of rhizosphere soil bacterial community, the abundance of dominant bacteria phylum, and dominant bacteria genus, climate characteristics and soil physical and chemical factors of Chinese seabuckthorn was revealed. RDA analysis of bacterial community α-diversity with soil physicochemical properties and climatic characteristics is presented in Figure 11a,b. The interpretation rates of soil bacterial community diversity, climate characteristics, and soil physical and chemical characteristics on the I and II axes were 46.11% and 5.01%, respectively, and the cumulative interpretation rate reached 51.12%. Among them, the arrow line of pH value is the longest, its explanation rate is 13.0%, and its contribution rate is 25.4%, which is significant (*p* = 0.016). Followed by ALT and SWC, the explanation rates were 8.8% and 7.2%, respectively, the contribution rates were 17.1% and 14.1%, respectively, and the *p* values were 0.03 and 0.04, respectively, both reaching significant levels. The study findings confirmed that among multiple environmental factors, soil pH serves as the primary ecological driver shaping the alpha diversity of Chinese hippophae rhizosphere microbial communities, while ALT and SWC constitute secondary influential factors. Collectively, these three factors synergistically determine the alpha diversity patterns of rhizosphere bacterial communities.

In the bacterial community, the RDA analysis between the horizontal abundance of the top five phyla and soil physical, chemical characteristics, and climate characteristics is shown in Figure 11c,d. The top five bacterial phyla in abundance, climate characteristics, and soil physical and chemical characteristics were explained by 41.65% and 16.38% on the I and II axes, respectively, and the cumulative explanation rate reached 58.03%. Among them, the arrow line of NORTH is the longest, its explanation rate is 16.7%, and its contribution rate is 23.8%, which is significant (*p* = 0.002). Followed by EAST and SEC, the explanation rates were 15.3% and 9.0%, respectively, the contribution rates were 21.8% and 12.0%, respectively, and the *p* values were 0.002 and 0.004, respectively, both reaching significant levels. The study findings confirmed that among multiple environmental factors, NORTH serves as the primary ecological driver for phylum-level abundance changes in Chinese hippophae microbial communities, while EAST and SEC constitute secondary influential factors. Collectively, these three factors synergistically shape the phylum-level abundance variations of rhizosphere bacterial communities.

The RDA analysis between the top five horizontal abundance of bacterial community genera, soil physical and chemical characteristics, and climate characteristics is shown in Figure 11e,f. The top five bacterial genera in abundance at the genus level, climatic characteristics, and soil physicochemical properties, exhibited explained variances of 23.77% and 17.40% on Axis I and Axis II, respectively, with a cumulative explained variance of 41.17%. Among them, the arrow line of SOM value is the longest, its interpretation rate is 13.3%, and its contribution rate is 25.0%, which is significant (*p* = 0.002). Followed by STP, the explanation rate was 6.9%, the contribution rate was 13.0%, and the *p* value was 0.052, which did not reach a significant level. The results show that among multiple environmental factors, soil SOM is the primary ecological factor driving the change of the horizontal abundance of the rhizosphere microbial community of Chinese hippophae microbial.

## 4. Discussion

### 4.1. Changes of Bacterial Community Structure and Function in Rhizosphere Soil of Chinese Seabuckthorn Under 13 Distribution Regions

As a core functional component of the rhizosphere soil ecosystem, rhizosphere bacterial communities play a pivotal role in maintaining soil health and promoting plant growth and development by driving biochemical processes involved in the cycling of carbon, nitrogen, phosphorus, and other essential elements [29,30]. In this study, wild Chinese seabuckthorn resources in 13 geographical distribution regions in the northwest arid area of China were taken as the research object, and rhizosphere soil samples were collected. By measuring soil physicochemical characteristics, combining with geospatial data and climate parameters, molecular biology technology and a high-throughput sequencing platform were used to deeply analyze the composition structure and functional characteristics of rhizosphere soil bacterial communities under different habitat conditions. The results showed that the number of bacterial operational taxonomic units (ASV) in S2 and S12 distribution regions reached 3072 and 3637, respectively, which was significantly higher than that in S11(2158) and S13(1984). This discrepancy may be closely associated with soil pH variations: the rhizosphere soils of S2 and S12 distribution regions exhibit neutral to alkaline conditions, whereas S11 displays acidity and S13 shows alkalinity. Neutral-alkaline soil environments tend to facilitate natural vegetation succession and colonization by symbiotic bacteria, thereby promoting bacterial population proliferation and subsequently increasing the number of ASV in its rhizosphere [10]. The abundance composition of rhizosphere bacterial communities in 13 Chinese seabuckthorn distribution regions reveals shared characteristics in dominant phyla and genera across different distribution areas. At the phylum level, Proteobacteria, Actinobacteriota, Acidobacteriota, Gemmatimonadota, and Chloroflexi constitute the core bacterial groups of Chinese seabuckthorn. At the genus level, *Achromobacter*, *RB41*, *Sphingomonas* spp., and *Methylotenera* form the core bacterial groups, except in habitats S6 and S11. These bacterial phyla and genera, serving as core members of the rhizosphere bacterial community in Chinese seabuckthorn, play multiple critical roles in ecosystems, with functions encompassing material cycling, energy flow, interspecific interactions, and environmental regulation [16,27]. However, bacterial communities still exhibit differences across distribution regions, particularly in the composition of dominant genera. In this study, the relative abundance of *Sphingomonas* spp. in the S11 distribution region was significantly lower than in the other 12 regions. This may result from the reciprocal preference selection between *Sphingomonas* spp. bacteria and soil pH [31]. This genus has a low tolerance threshold for saline–alkaline stress, and its population density is negatively correlated with soil pH. Consequently, in neutral-alkaline soils (pH ≈ 8.0), the relative abundance of *Sphingomonas* spp. increases significantly [32]. Research indicates that *Sphingomonas* spp. has functions such as improving acidic soils and accelerating available nutrient cycling; the low abundance of *Sphingomonas* spp. in the S11 distribution region further contributes to the strong soil acidity and low SAK content in this area [31,32].

This study showed that the diversity of rhizosphere soil bacterial community of Chinese seabuckthorn in 13 geographical distribution regions had significant spatial heterogeneity. Specifically, compared with S11, the Shannon index, Ace index, Chao1 index, and the number of OTU in the other 12 distribution regions, especially S5 and S12, were significantly increased. This aligns with the findings of Ilham et al. [33] and Guo et al. [34], indicating that climatic characteristics and soil physicochemical properties significantly influence soil bacterial alpha diversity. Specifically, habitats with lower elevation, neutral to alkaline soil pH, and higher nutrient content exhibit stronger soil bacterial alpha diversity, as reflected by higher Shannon index, Ace index, Chao 1 index, and OTU richness. According to PCoA analysis, the composition and structure of soil bacteria (β diversity) in different distribution regions have different characteristics. Among them, S6 and S11 have low aggregation, while the other 11 regions have relatively high aggregated soil bacterial composition. There are differences between S11 and other regions in the rhizosphere microbial community structure of Chinese seabuckthorn. This effect may be due to the differences in soil physicochemical characteristics (such as pH, water content, and soil nutrients), or it may be related to climate characteristics [21].

The construction and visualization analysis of the rhizosphere microbial ecological network for Chinese seabuckthorn enabled effective mapping of microbial interactions and community stability within the plant rhizosphere [35]. In this study, the collinear network analysis of microorganisms was used to predict the core taxa in microbial communities, which play an important role in the related networks and functions of plant rhizosphere soil systems. For example, core group microorganisms can protect plants from pathogens in agricultural production or promote the interaction between microorganisms to strengthen the ability of plants to resist adversity stress [36,37]. It was found that the rhizosphere soil bacterial network of Chinese seabuckthorn was complex, with 950 nodes and 17,196 edges, and the positive correlation was as high as 88.19%. This indicates that the vast majority of bacterial taxa form stable cooperative relationships through mechanisms such as mutualism and synergistic coexistence. This interactive mode not only optimizes the efficiency of rhizosphere nutrient circulation but also enhances the ability of plants to cope with environmental stress by enhancing the functional complementarity among microorganisms [38]. Further investigation revealed that *Proteobacteria* (27.81%), *Acidobacteriota* (21.87%), and *Actinobacteriota* (17.20%) demonstrated high connectivity, centrality, and abundance, occupying central positions within the network. These phyla represent the most critical bacterial groups in the rhizosphere soil of Chinese seabuckthorn in the arid regions of Northwest China. They play a pivotal role in driving the stability of bacterial community structure and function, improving soil conditions, and enhancing plant productivity [39,40,41].

Cluster analysis revealed a distinct bimodal differentiation pattern among the rhizosphere bacterial communities of Chinese seabuckthorn across the sampled regions: populations from S10 and S12 clustered into Group 1, while the remaining 11 regions formed Group 2. This spatial heterogeneity differentiation model may be derived from two dimensions of ecological driving factors: one is the micro-habitat screening formed by the difference of vegetation–soil complex characteristics in different distribution regions [42], and the other is the macro-environmental constraints caused by the spatial differentiation of regional climate characteristics [21]. Meanwhile, Guo et al. [34] found that changes in soil bacterial community structure can lead to differences in soil bacterial functional characteristics. In this study, we predicted the rhizosphere soil bacterial functions of Chinese seabuckthorn based on the PICRUSt functional classification and found that the soil bacterial functions of the 13 distributions were significantly different, which was consistent with their findings. In the functional hierarchy analysis, Metabolism and Genetic Information Processing are the core functional modules in all distribution regions. This functional convergence can be attributed to the evolutionary adaptation of microbial survival strategies, i.e., metabolic functions serve as the basic platform for energy–matter conversion, sustaining the basic life activities of bacterial communities [42]; and genetic information processing functions drive bacterial community adaptations and innovations through the regulation of gene expression networks, which synergistically endow Chinese seabuckthorn rhizosphere soil bacteria with a central position in the soil ecosystem [43]. Notably, the S11 population exhibited marked functional deviation, with significantly reduced metabolic activity compared to other regions. This metabolic suppression may result from the low soil pH (6.46) in S11, as acidic conditions can comprehensively impair bacterial metabolism through direct toxic effects, resource limitation, and energy metabolism disruption, ultimately inhibiting bacterial growth and reproduction [44,45,46]. The S10 distribution region also exhibited significant functional deviation, with markedly reduced intensities of Energy_Metabolism and Translation compared to other regions. The reason may be that the soil moisture content in S10 is less than 5%, which is substantially lower than in the other 12 distribution regions. The arid soil environment in S10 severely inhibited the performance of Energy_Metabolism and Translation processes in soil bacteria [27,39,46].

### 4.2. Correlation Analysis of Bacterial Community Diversity in Rhizosphere Soil of Chinese Seabuckthorn with Soil Physicochemical Characteristics and Climate Characteristics

Based on Mantel test correlation analysis, this study elucidated the interaction patterns among soil–microbe–climate systems in the rhizosphere of Chinese seabuckthorn in the arid regions of Northwest China. The results show that there is a significant coupling relationship between soil physicochemical characteristics (SOM, STN, STP, STK, pH, SEC) and climatic and geographical factors (longitude, latitude, altitude, annual average temperature, and annual precipitation). Notably, soil organic matter (SOM) and total nitrogen (STN) content exhibited highly significant negative correlations with pH, likely due to alkaline conditions inhibiting organic matter decomposition enzyme activity and nitrogen mineralization processes [44,45,46,47,48]. Soil total phosphorus (STP) content showed an extremely significant negative correlation with altitude (ALT), which may be attributed to the dual mechanisms of ALT not only limiting the activity of rhizosphere microorganisms but also hindering the development of plant roots, resulting in the weakening of phosphorus activation [49]. Further analysis demonstrated significant associations between bacterial community α-diversity indices (OTU richness, Shannon index, Pielou index, Chao1 index) and soil physicochemical characteristics and climate characteristics. Specifically, α-diversity metrics displayed significant (*p* < 0.05) or highly significant (*p* < 0.01) correlations with SOM, STN, STP, and pH. These findings suggest that Chinese seabuckthorn populations in the arid regions of Northwest China establish a self-reinforcing ecological feedback loop through “plant–soil–microbe” interactions. This system enables bidirectional enhancement of soil fertility improvement and bacterial community optimization, ultimately forming a sustainable ecological cycle [50].

The diversity of rhizosphere bacterial communities in Chinese seabuckthorn in the arid regions of Northwest China is jointly regulated by soil physicochemical factors and climatic characteristics, with soil pH emerging as the core driving factor. Research indicates that pH exerts its influence through three interconnected pathways: (1) When soil pH falls within the neutral to slightly alkaline range, it directly stimulates the proliferation of symbiotic bacterial groups such as Rhizobia, thereby driving bacterial diversity formation [51]; (2) This pH condition enhances cellulase and protease activities, accelerating carbon cycling efficiency and indirectly strengthening bacterial community diversity and stability through expansion of the soil nutrient pool; (3) Neutral to slightly alkaline environments facilitate ecosystem natural restoration processes, promoting systematic expansion of associated symbiotic bacterial populations and ultimately enriching bacterial community structure [52]. This process reveals that pH value not only directly affects bacterial community through soil fertility parameters but also indirectly regulates the diversity and stability of bacterial community through plant–microorganism interaction network [53]. During ecological restoration projects involving Chinese seabuckthorn cultivation, regulating soil pH to the optimal range of 7.8–8.2 can significantly enhance carbon cycling functionality and nutrient transformation efficiency mediated by rhizosphere bacteria. The influence of altitude (ALT) and soil water content (SWC) on bacterial community diversity in rhizosphere soil of Chinese seabuckthorn is second only to soil pH value. ALT indirectly affects bacterial diversity through its impacts on soil nutrients and vegetation diversity, whereas SWC directly shapes bacterial community structure through water stress. These findings corroborate conclusions from previous studies by Guo et al., forming a robust interdisciplinary validation [21].

## 5. Conclusions

(1) Among the rhizosphere soil bacterial communities of Chinese seabuckthorn in 13 distribution regions in the northwest arid region of China, the number of bacteria ASV in S2 and S12 distribution regions is the highest, with 3072 and 3637, respectively, while that in S11 distribution area is the least, with only 1358; there are significant differences in α diversity indexes among 13 distribution regions: the number of OTU, Shannon index, Ace index, and Chao1 index in S12 distribution area are the highest, which are significantly higher than those in S11 distribution area.
(2) The phyla *Proteobacteria*, *Actinobacteriota*, *Acidobacteriota*, *Gemmatimonadota*, and *Chloroflexi* were identified as the core bacterial taxa in the rhizosphere soil of Chinese seabuckthorn.
(3) Metabolic functions and genetic information processing functions dominated across all distribution regions, with significantly higher abundance of these functions in the S10 compared to the S11 region. Bray-Curtis distance-based clustering grouped the bacterial communities into two distinct clusters: S10 and S12 formed Group 1, while the remaining regions constituted Group 2.
(4) Redundancy analysis (RDA) indicated that soil pH was the primary environmental factor shaping the microbial community α-diversity in the rhizosphere of Chinese seabuckthorn, followed by altitude (ALT) and soil water content (SWC) as secondary influencing factors.

In summary, this study systematically elucidated the biogeographical patterns of rhizosphere microbial communities across 13 typical distribution regions of Chinese seabuckthorn in the arid regions of Northwest China. The findings reveal significant variations in community composition, α/β-diversity, and functional traits. These results provide a microbiological foundation for understanding the growth and development of Chinese seabuckthorn populations and screening beneficial microorganisms. Additionally, the study offers theoretical support for the ecological restoration applications of this native tree species in high-altitude arid environments. In future research, we will incorporate metagenomic sequencing to more precisely resolve functional genes and plan to conduct isolation, identification, and evaluation of growth-promoting effects for functional strains.

## Figures and Tables

**Table 1:** Geographic information and climatic characteristics of the collection site.

Number	Sampling Location	East/°	North/°	Altitude (ALT)/m	Air Pressure (ATM)/KPa	Average Annual Temperature (AAT)/℃	Average Annual Rainfall (AAR)/mm
S1	Huzhu Tuzu Autonomous County	101.96	36.84	2696.31	71.39	1.72	126.09
S2	Minhe Huizu and Tuzu Autonomous County	102.83	36.32	2644.83	73.94	5.46	101.83
S3	Ledu District	102.83	36.33	2753.57	72.66	1.72	126.09
S4	Qilian County	100.25	38.18	2920.81	71.50	−0.02	124.21
S5	Banma County	100.87	32.71	3441.00	61.22	1.93	158.83
S6	Wulan County	99.08	36.79	3159.06	70.10	1.36	76.41
S7	Huangyuan County	101.26	36.68	2923.11	71.71	1.76	122.61
S8	Gonghe County	100.62	36.28	2947.60	71.39	3.47	102.03
S9	Tongren City	102.02	35.52	2387.93	74.40	5.53	114.66
S10	Yushu City	97.01	33.01	3665.00	65.80	1.93	132.35
S11	Zhang County	104.22	34.72	2725.20	73.26	4.51	124.83
S12	Urumqi County	87.36	43.69	1562.50	82.73	3.72	63.72
S13	Qinghe County	90.38	46.68	1230.00	86.43	2.21	29.04

**Table 2:** Physical and chemical properties of soil in sampling area.

Number	SOM/(g/kg)	STN/(g/kg)	STP/(g/kg)	STK/(g/kg)	SAN/(mg/kg)	SAP/(mg/kg)	SAK/(mg/kg)	SWC/(%)	pH	SEC/(ms/cm)
S1	56.77	3.05	0.75	15.49	225.55	6.74	39.07	14.52	7.60	0.580
S2	26.70	1.79	0.64	19.05	106.05	2.10	67.98	11.09	8.07	0.547
S3	95.35	5.97	0.99	20.60	413.27	2.85	157.39	32.74	7.53	0.623
S4	41.77	2.55	0.63	18.34	181.03	1.20	47.38	7.21	7.99	0.563
S5	42.65	2.61	0.59	17.95	198.99	3.07	21.16	23.88	7.65	0.683
S6	9.04	0.90	0.56	18.33	46.43	3.70	87.48	4.22	7.92	0.603
S7	38.03	2.14	0.75	16.08	157.39	7.98	85.09	16.96	8.39	0.663
S8	22.73	1.65	0.59	17.97	98.34	1.25	215.70	17.63	6.85	0.613
S9	40.60	2.22	0.68	17.21	124.07	2.70	139.98	7.44	8.05	0.603
S10	27.46	1.83	0.53	18.27	127.15	6.11	90.20	3.72	8.08	0.513
S11	98.58	5.00	1.17	18.55	397.37	3.39	42.63	28.40	6.46	0.603
S12	49.16	3.30	0.79	19.76	232.10	7.16	153.40	7.22	7.89	0.617
S13	9.45	0.77	1.25	18.39	29.04	5.73	144.20	8.25	8.31	0.537

Note: SOM: Soil organic matter; STN: Soil total nitrogen; STP: Soil total phosphorus; STK: Soil total potassium; SAN: Soil alkali-hydrolyzable nitrogen; SAP: Soil available phosphorus; SAK: oil available potassium; SWC: Soil water content; SEC: Soil electrical conductivity.

## Data Availability

The raw sequencing data generated during this study has been deposited in the NCBI Sequence Read Archive (SRA) under BioProject ID PRJNA1297643. These datasets are publicly available and can be accessed via 39 SRA accessions, including SRR34734242, SRR34734241, SRR34734230, SRR34734219, SRR34734209, SRR34734208, SRR34734207, SRR34734206, SRR34734205, SRR34734204, SRR34734240, SRR34734239, SRR34734238, SRR34734237, SRR34734236, SRR34734235, SRR34734234, SRR34734233, SRR34734232, SRR34734231, SRR34734229, SRR34734228, SRR34734227, SRR34734226,SRR34734225, SRR34734224, SRR34734223, SRR34734222, SRR34734221, SRR34734220, SRR34734218, SRR34734217, SRR34734216, SRR34734215, SRR34734214, SRR34734213, SRR34734212, SRR34734211 and SRR34734210.
